# Academic Gender Disparity in Orthopedic Surgery in Canadian Universities

**DOI:** 10.7759/cureus.7205

**Published:** 2020-03-08

**Authors:** Toshimitzu Yue, Faisal Khosa

**Affiliations:** 1 Medical Education and Simulation, Vancouver General Hospital, Vancouver, CAN; 2 Radiology, Vancouver General Hospital, Vancouver, CAN

**Keywords:** orthopedics, surgeons, gender disparity, h-index

## Abstract

Introduction

Academic medicine is notorious for being "male-dominated." We hypothesized that there were significant and quantifiable levels of gender disparity in academic orthopedic surgery, and this article attempts to quantify the extent of the existing disparity. Also, we examined the research productivity of academic faculty in orthopedic surgery and its correlation with academic ranks and leadership positions.

Methods

Our study design was cross-sectional in nature. We searched the Canadian Resident Matching Service (CaRMS) to compile a list of medical schools that offer orthopedic surgery training for residency. A total of 713 academic orthopedic surgeons met our inclusion criteria. Of the 713 orthopedic surgeons, 518 had an H-index score available on Elsevier’s Scopus (Elsevier, Amsterdam, Netherlands). The gender, academic rank, leadership position, and H-index were compared. Data analysis was done with Statistical Package for the Social Sciences (SPSS; IBM, Armonk, NY). The binomial negative regression was used to compare the average H-index between men and women at each rank.

Results

Our study results reveal that academic orthopedic surgery in Canada is male-dominated, with men holding 87% of the academic positions. Female academic orthopedic surgeons held lower academic ranks, such as assistant professor or lecturer. Women orthopedic surgeons had lower H-index scores compared to their counterparts in ranks above the assistant professor. Our findings imply that research productivity and the ratio of average H-index scores comparing men to women (HM/HF) grow larger with each academic rank. At a 90% confidence level, women were less likely to hold leadership positions than men at an odds ratio (OR) of 0.52 [90% confidence interval (CI): 0.29-0.925, p: 0.03]. There were no significant differences in H-index between men and women for departmental leadership positions.

Conclusion

Women were underrepresented in number, rank, and academic productivity (H-index). We offer possible factors that may have contributed to this finding as well as potential solutions.

## Introduction

In 1988-1989, the Canadian Post-M.D. Education Registry (CAPER) census showed that 34% of medical graduates in Canada were female. In the census of 2017-2018, the number of female graduates had grown to 56%, indicating that the proportion of women in medical schools had increased significantly over the last 30 years. However, recent research has shown that subspecialties of radiology, psychiatry, and dermatology still remain male-dominated [1-5]. Currently, only 0.6% of female graduates choose orthopedic surgery residency in America and only 7.6% of orthopedic surgery residents are women [6,7]. In Canada, only 11% of working orthopedic surgeons were women [8].

It is known that most surgical specialties are male-dominated. Previous investigations in radiology were able to quantify the disparity and provide strong evidence to suggest that research productivity was a major factor for the ongoing disparity [1-3]. These studies found that women were a minority in the academic practice, were at the lower rungs of academic ranks, were disproportionally represented in leadership positions, had lower research productivity and lower H-index scores when compared to men. Similar results were also found in psychiatry and dermatology [4,5]. This suggests that not only is the number of women in a specialty the marker for equality and equity but also their ability to progress academically.

Our literature search did not reveal any previous publications on the role of academic productivity on gender disparity in academic orthopedics. It would be imperative to quantify and determine if women in orthopedic surgery are faced with challenges shown in other specialties. We, therefore, investigated the presence and extent of gender disparity in academic orthopedic surgery and its relation to academic productivity.

## Materials and methods

Procedure

Our methodology has been validated in several recent publications. [1-5,9] We obtained a list of orthopedic programs to gather the academic faculty members of each orthopedic department. The Canadian Resident Matching Service (CaRMS) website enlists a total of 17 university hospitals that offer orthopedic training programs. Each university website provided a full faculty list of their department, and the only exceptions were the University of Montreal and the University of Laval. The administrative departments of these two universities were contacted to obtain their faculty listing. All faculty listings included academic rank and leadership roles. Any missing or outdated information was cross-referenced with online sources such as faculty newsletters, LinkedIn (LinkedIn, Mountain View, CA) and Google (Alphabet Inc., Mountain View, CA). The data collection period ran from December 2018 to March 2019.

The inclusion criteria were faculty members with academic ranking (i.e., professor, associate professor, assistant professor, and lecturer), an MD degree or equivalent, and those listed as a faculty member on the university website or orthopedic department faculty list. The hierarchy of academic rank in ascending order was as follows: lecturer, assistant professor, associate professor, and professor or full professor. Chair, department head, chief, vice-chair, and program director were considered leadership roles.

The exclusion criteria were adjunct and retired faculty members, those without an MD degree or equivalent, those without Scopus records, and those whose gender could not be identified. We used Elsevier's Scopus (Elsevier, Amsterdam, Netherlands) for gathering the data about the publications, H-index, citations, and years of research. Scopus was chosen as the database as it is the most reliable tool to measure the H-index and is more consistent when compared to Google Scholar (Alphabet Inc., Mountain View, CA) or Web of Science (Clarivate Analytics, Philadelphia, PA) [10,11].

Analysis

The H-index had the highest predictive value for research productivity when compared to the number of citations, number of papers, or mean citations per paper [12,13]. H-index is a compound of the number of publications and the average number of citations per publication from the time of first publication. The differences in H-index scores between men and women were calculated as a ratio of average H-index score comparing men to women (HM/HF). To determine if there is a significant difference between H-index in each academic rank and leadership position, data was run through Statistical Package for the Social Sciences (SPSS; IBM, Armonk, NY) and a negative binomial regression model to generate the ratio of average H-index between men and women. Those with no publication or only one publication were given an H-index of 0 and were not included in the negative binomial analysis. We calculated the ratio of average for each academic rank as well as those with a leadership position and without leadership position.

## Results

Academia

Men occupied most of the academic positions in academic orthopedic departments (Table 1). These numbers represent the entirety of orthopedic academic professionals in Canada. For this table, we included those without the H-index score. Of the 713 academic positions, women held 13% of these positions. Also, most women held lower academic ranks such as assistant professors and lecturers (78%). The majority of the men held academic ranks of assistant professor and above (73%).

**Table 1:** Gender distribution in academic rank

Gender	Lecturer, %	Assistant, %	Associate, %	Professor, %	Total, %
Female	5.7	4.7	2	1.1	13.3
Male	23.3	31.7	18	13.7	86.7

We show a comparison of the ratio of average H-index scores between men and women (Table 2). Those without H-index were excluded for this analysis and 518 individuals met this criterion; 68 were female and 450 were male. There was no significant difference in the H-index score between men and women at the rank of lecturer (HM/HF = 1.25, 95% CI: 0.84-1.87, p: 0.27). A significant difference between the H-index scores was found when comparing men and women at the rank of assistant, associate, professor, and the overall average. When calculating the ratios, men held a higher H-index score as an assistant (HM/HF = 1.50), associate (HM/HF = 1.66), and full professor (HM/HF = 2.59). 

**Table 2:** Ratio of average H-index between men and women in each academic position HM/HF: ratio of average H-index score comparing men to women; CI: confidence interval The negative binomial regression model was used to generate the ratio of average H-index between male and female orthopedic academics for each academic position. Only faculty members with an H-index of >0 were included in this set

Academic position	Ratio of average value (HM/HF)	95% CI	P-Value
Lecturer	1.25	[0.84-1.87]	0.27
Assistant	1.50	[1.08-2.10]	0.02
Associate	1.66	[1.04-2.63]	0.03
Professor	2.59	[1.29-5.21]	0.01

Leadership

Overall, men held more leadership positions than women (15.9% and 1.5% respectively) (Table 3). Of all the female academic orthopedic surgeons, around 10% held leadership positions. Of all the male orthopedic surgeons, nearly 20% held leadership positions. The odds ratio (OR) of being a woman holding a leadership position was 0.52 (90% CI: 0.29-0.925, p: 0.03) compared to men.

**Table 3:** Gender distribution in leadership position

Gender	Leadership, %	Non-leadership, %
Male	15.9	70.7
Female	1.5	11.9

Men on average held higher H-index score, regardless of whether they held a leadership role or not, where HM/HF was above 1 (Table 4). Men with leadership roles had a significantly larger H-index score than women with the ratio of an average H-index score of 2.49 (95% CI: 1.31-4.73, p: 0.005). There was no significant difference between H-index score between those with or without leadership roles.

**Table 4:** Ratio of average value between male and female orthopedic academics in leadership positions HM/HF: ratio of average H-index score comparing men to women; CI: confidence interval The negative binomial regression model was used to generate the ratio of average H-index between male and female orthopedic academics for holding leadership positions. Only faculty members with an H-index of >0 were included in this set

	Ratio of average value (HM/HF)	95% CI	P-value
Leadership-role	2.49	1.31-4.73	0.005
Non-leadership role	1.97	1.37-2.83	0.0003

## Discussion

Our study found that there is a significant gender disparity in academic orthopedics in Canada, with men holding most of the higher academic as well as leadership positions. Previous research has found that there are at least six common factors for gender disparity in orthopedic surgery: a perceived hostile work environment; lack of equality in rank and leadership by gender; a disparity in salary; an unfavorable work-life balance for women compounded by the disproportionate burden of family responsibilities; a lack of physical strength; and a lack of strong mentorship in school [14-16]. The current study investigated the holistic outcome of these factors and measured it through research productivity (H-index), leadership positions, and the academic rank of academic orthopedic surgeons.

Disparity in academic rank and leadership positions by gender

Most women (79%) were at the lower academic ranks of either lecturer or assistant professor. Male and female lecturers did not have any significant difference in their H-index score (HM/HF = 1.25, 95% CI: 0.84-1.87). Our data show that this disparity becomes progressively evident at the rank of assistant professor and beyond.

Higher academic rank and research may hold a synergistic relationship in which higher academic rank provides increasing opportunities and support for research through networking with other professionals, access to grants, collaboration, and partnership. Our results show the differences in H-index scores between the two genders increased with ascending academic ranks. This suggests that men are securing the benefits of higher academic rank substantially more than women. Also, as academic rank ascends, the proportion of women holding higher ranks drop. Only 1.1% of full professors were women.

Leadership positions were also male-dominated but were not significantly associated with H-index. However, the proportion of women holding a leadership position was lower than men (OR: 0.52). Unfortunately, we could not find a significant difference in OR using a 95% CI due to a smaller sample size of women in a leadership position.

The results of our study suggest that orthopedics is a subspecialty in which gender disparity exists both in leadership positions and academic ranks. Besides, we found that women had a lower H-index score. H-index and research productivity are strong predictors for academic promotion [12]. Our results show that research productivity is a major factor in the disparity in academic orthopedic surgery.

Another study found that women represented only 30.4% of psychiatry Journals' editorial board members [9]. This study found that although women held positions in a lower number of publications, these held equal value in impact factors. Hence, the study concluded that though gender disparity existed there was no gender bias in these editorial boards. In our study, we did not evaluate the impact factors for each publication. Thus, we cannot make any conclusion on gender bias or systemic oppression of a group. Academic psychiatry in the United States is also a male-dominated specialty and showed similar trends to our study: women held lower H-index scores and were at lower academic positions [4].

Disparity in salary

Prior research has shown that orthopedic surgery has one of the highest adjusted salary differences between men and women ($40,953, 95% CI: $2277-$79,628])[16]. Additionally, female academic physicians earn much less than men. Women earned $141,325 (p: 0.5, 95% CI: $135,607- $147,043) and men $172,164 (p: <0.5, 95% CI: $167,357-$176971) [17]. Surprisingly, across all subspecialties, despite gaining academic rank and promotions, women continue to suffer from the wage gap (−$485 per year of seniority; p: 0.01) [18]. Women receive a lower salary and obtain fewer promotions within their ranks than men. This could be a strong disincentive for women to pursue an academic career and a reason for our findings. Women are required to work harder for promotion; however, that promotion is fiscally not as rewarding when compared to that of men.

There is a significant wage gap between mothers and non-mothers, which is greater than the wage gap between men and women, suggesting that there are negative stereotypes associated with motherhood. Professional mothers are "mommy tracked", where they may be passed over for promotions and work opportunities when compared to non-mothers [19,20]. Also, women are aware of these stereotypes and prejudice, therefore making work-life balance a strong indicator and deterrent for choosing orthopedic surgery as a career [20]. 

Unfavorable work-life balance

The perception of work-life balance is a serious concern for women when selecting specialties such as orthopedic surgery [20]. In a survey of orthopedic faculty members and residents, married members were found to have lower emotional exhaustion (p: <0.0003) and higher personal achievement (p: <0.0001). However, 88% of the residents and 92% of the faculty members were males [21]. A reasonable interpretation of these findings is that married male orthopedic surgeons tend to have a more favorable work-life balance than their non-married male colleagues. This is in stark contrast to women, who may have to choose between family and career. In orthopedic surgery, men were more likely to be married (57.8% vs. 37.9%, p: <0.001) and have children (31.5% vs. 12.0%, p: <0.001) than women [22]. It is evident that societal gender-roles (women as caregivers) can be a factor for less interest in orthopedic surgery. It is also imperative that we consider how work-life balance affects female orthopedic surgeons. Taking up higher academic ranks, leadership roles, and research projects requires an additional time commitment to work and hence time away from caregiving.

Hostile work environment

It was found that female medical students did not apply to orthopedics residency due to the potential of a hostile work environment [13]. A 2016 study found that women are three times more likely to leave orthopedic residency compared to men and that sexual harassment and sex discrimination are major factors in this decision [23]. Another study found that one in three women in academic medicine reports having personally experienced sexual harassment [24]. Among these women, 40% described experiencing coercive advances or threats to engage in sexual behavior; 47% reported that these experiences negatively affected their career advancement. These findings do support the outcomes of our study.

Solutions

Our study found two potential sources of disparity that could be addressed. First is the low number of women entering into orthopedic surgery. To remedy this, female medical students could be exposed to more elective orthopedic experience. When compared to men, women were more likely to pursue orthopedic surgery after clinical exposure in their rotation or elective [25]. Also, women felt that their peers entered orthopedic surgery due to greater acceptance by senior faculty in that field [25].

The second source of disparity is the overall lack of promotions in academic medicine. This has been predominantly attributed to significantly lower research productivity by female orthopedic surgeons. Research mentoring of physicians during residency was found to correlate with an increased pursuit of research and success in academic medicine [14]. However, women felt it was harder for them to find mentors compared to their male colleagues due to their gender [26]. A pilot study at the Indiana University School of Medicine found that 80% of individuals who participated in a mentorship program successfully published a manuscript. This survey suggested that mentorship increases research productivity through increased opportunity, professional and personal development such as research proficiency, interpersonal skills, and career development skills [27].

Limitations

Our study has its share of limitations. There was an inherent degree of potential inaccuracy in obtaining faculty listing from institutional websites, as these may not be updated in real-time. Another limitation was the use of Elsevier’s Scopus, even though it was deemed to be the most accurate and up to date source [12,13]. Finally, as Hirsch (2005, 2007) points out, there are major limitations to the H-index. H-index does not consider the impact score of publications but rather focuses on citations and the number of publications. For example, a Noble Prize winner with two publications and a high number of citations can have a similar H-index score to another individual with high publication numbers and low citations. Furthermore, the H-index score cannot differentiate between the primary investigator, research assistant, and first author. Credibility for work can be lost, leading to lower H-index caused by a change in institutional affiliation or name change. Women are more likely to change their names after marriage than men. Convenience sampling is open to bias, and information on websites may be outdated if not updated frequently. The H-Index does not discriminate between author order, differentiate between many papers of poor quality and one paper of good quality, or discriminate between self-citations [28]. Because years of active research are often determined by an author's year of first publication, those who take prolonged breaks during their careers may have their years of active research overestimated, a finding that may disproportionately affect women. This study is a cross-sectional study and does not capture the current trend indicating positive changes toward equality. Overall, in Canada, female orthopedics are found to be significantly younger than their male counterparts (50% of the women are between the ages of 35-45; 41% of male orthopedic surgeons are 55 years of age or older) [29].

## Conclusions

Our study documents the extent of and reasons for disparity in academic orthopedic surgery across Canada. Women are a minority in academic orthopedic surgery, occupy lower academic ranks, and are disproportionately represented in leadership positions. Research has shown that exposure in medical schools to orthopedic surgery and the availability of mentors and role models can help recruit and retain women in academic disciplines. This study is a cross-sectional study and cannot capture the trend. We hope that this study can be used as a baseline for future studies that monitor gender equality in orthopedic surgery in Canada.

## Appendices

Disclosures and conflict of interest: Dr. Khosa is the recipient of the Canadian Association of Radiologists - Young Investigator Award (2019); Rising Star Exchange Scholarship Program of French Society of Radiology (2019) and Humanitarian Award of Association of Physicians of Pakistani Descent of North America (2019). The authors did not receive financial support for this study from any for-profit or non-profit organizations. The authors did not have any relationship with any organization or individuals that may have influenced this study.
